# The Role of Snow-Related Environmental Variables in Plant Conservation Plans in the Mediterranean Mountains

**DOI:** 10.3390/plants13060783

**Published:** 2024-03-10

**Authors:** Jose A. Algarra, Paloma Cariñanos, María M. Ramos-Lorente

**Affiliations:** 1 Department of Botany, University of Granada, 18071 Granada, Spain; 2 Botanic Garden Hoya de Pedraza (Sierra Nevada), The Environment and Water Agency of Andalusia, 18014 Granada, Spain; palomacg@ugr.es; 3 Andalusian Institute for Earth System Research (IISTA-CEAMA), University of Granada, 18071 Granada, Spain; 4 Department of Sociology, Faculty of Health Sciences, University of Granada, 18071 Granada, Spain; mdmrl@ugr.es

**Keywords:** Mediterranean high summits, snowpacks, Sierra Nevada National Park, adaptive management, seedlings, Plant Restocking Project

## Abstract

This study aims to analyze the effects that snow cover may have on the survival of one-year-old seedlings from 15 different taxa in the Mediterranean high mountains (Sierra Nevada National Park, SE Spain) in order to have clearer criteria for the planning and management of restoration efforts in these environments. Additionally, the influence of variables that have been scarcely explored up to now is also revised. We use the survival rates of the seedlings observed from the ecological restoration trial as reference values. The survival data analyzed are based on six variables to evaluate their effects. The results confirm that the permanence of snow is a favorable factor for seedlings, independent of the plant community. Contrastingly, a specific type of foundation (stones and rocks) stands out for being clearly unfavorable, regardless of other variables. For both altitude and solar radiation, a worsening of the survival ratio has been observed as they increase. The species’ geographic ranges are all shown to be unfavorable for taxa of a boreo-alpine distribution. Finally, the plant community does not have a significant influence on the survival of seedlings. These results provide novel indications to improve the results of the first stages of restoration work in the Mediterranean high mountains. They are also valuable for the management and cataloging of threatened flora, as well as having direct applications in recovery plans and protection lists.

## 1. Introduction

Among the current changes in environmental conditions, climate warming presumably has the greatest potential to change species communities and distributions in high-mountain areas [1,2]. Numerous studies have found proof of recent changes in the composition of plant communities in mountainous zones [3,4,5,6,7,8,9,10,11], demonstrating changes in species distribution throughout the altitudinal range as a consequence of the effects of climate change. In many plant communities, substitutions by species from warmer habitats have been observed, especially at lower altitudes [12]. The term “thermophilization” is applied to the phenomenon by which the number of species adapted to warmer temperatures increases while that adapted to lower temperatures declines [8]. In some cases, such as in the Mediterranean high mountains, this has led to a decline in species diversity [7,13]. This scenario makes it necessary to know what species and communities will be the most affected so that effective corrective strategies can be designed.

Ecological degradation in mountain systems is a widespread process globally, and, in these very exclusive and fragile habitats, it becomes a serious threat to be taken into account. Among the main causes of threat, we can highlight not only those of anthropogenic origin, such as the management of hunting, livestock, mountaineering, growing tourism, and lack of environmental awareness [14], but also those due to climate change (increased temperatures and decreased precipitation).

In the current scenario of accelerated global change, there is an urgent need to optimize resources to obtain favorable results in plant conservation and restoration. There are many factors that may make this work more difficult, perhaps the most important being those related to the specificity of the species’ habitat, inefficient reproductive biology, topographic inaccessibility, interrelationships with other species (mainly fauna), etc. With these obstacles in mind, it is invaluable to have a minimum amount of information to guide future efficient restoration actions. With proven knowledge, the human and economic efforts invested are infinitely more profitable. This entire process is accelerated in a scenario in which higher temperatures and less precipitation are expected in many mountain systems [15,16].

Usually, variables such as slope, target plant community, plant cover, orientation, etc., have already been taken into consideration in plant restoration programs [17,18,19]. Many of them give generic favorable (or very favorable) responses regardless of the taxon in question [17,18]. Thus, in the case of plant reforestation carried out with *Pinus nigra* and *P. sylvestris* in Sierra Nevada (Spain), it was observed that both survived better when they were planted under and on the north side of spiny shrubs, while mortality was higher in open areas [17,18]. The facilitative effect of shrubs as nurse plants has also been demonstrated in low-altitude areas and sunny, drier slopes of the Mediterranean area [18]. On other occasions, drought stress in summer was the main cause of *Silene ciliata* mortality, even though germination occurred immediately after snowmelt [19]. However, in mid-to-high-elevation mountainous zones, the effect of snow and other climate factors such as wind or insolation also has a prominent role in plant establishment and growth [20,21], and thus could mark the difference between a successful project or generalized failure [22,23]. Regarding the permanence and amount of snow, the limited literature shows success or failure depending on each taxon in particular. Therefore, a generic answer is not obtained in the cases studied in arctic–alpine areas [20,21], and it is necessary to delve deeper into this study in the Mediterranean high mountains.

Regarding the early life history stages (germination, seedling, etc.) of alpine or subalpine species, Europe is the place where the most studies have been carried out [24]. The most researched variables, in order of frequency, include the following: temperature, snow cover, water availability, nutrient availability, and post-fire responses. Temperature is, by far, the most used variable, especially in the context of global warming. On the other hand, the least studied is post-fire responses, and nutrient or water availability. The number of studies related to seed germination in the natural environment and the soil seed bank is very deficient. However, changes in snow cover duration are some of the most evident effects of climate change at high altitudes [25], and although they exist, the number of studies on snow cover is not large, while the number of studies that are directly focused on restoration is even smaller. Previous work [24] seems to indicate that early snowmelt could facilitate the first stages, while high radiation, which would further exacerbate the effects of drought, seems to be negative for the survival of seedlings.

In the Mediterranean high-mountain summits, temporary persistence of snowpacks after the winter season (the so-called “neveros” in southern Spain) is frequently observed. This, coupled with observations of frozen seedlings in nurseries, located at 600 m, of species accustomed to living on peaks between 2600 and 3000 m in winter led us to suspect that the residence time of snow may be a determining factor in seedling survival, since their coverage spans extremely low temperatures and harsh winds [26,27]. In the Mediterranean region, this aspect becomes even more decisive as a result of the long periods of drought characteristic of this bioclimate, which has been exacerbated in recent decades [28], where each year it is scarcer and remains for less time. Taking into account that this can have a significant impact on snow cover, which tends to be a shrinking resource [29,30], one of the main problems with this is its effect on the recruitment of new seedlings. This has already been observed in some species present in high mountains, such as *Pinus sylvestris* L. in boreal environments of Finland [31]. Given the possibility that this could be extended to other species in these habitats, this variable will have a crucial role in future restoration efforts and in the management of plant communities in mountainous zones in the new expected climate scenario [32,33]. It is therefore necessary to know how and to what extent snow cover duration affects plant survival in order to adequately plan management strategies.

Sierra Nevada (SE Spain, 37° N, 3° W) is one of the most outstanding massifs of the Mediterranean region (reaching 3479 m). This well-known fact has been proven by famous botanists since ancient times, distinguishing two or three clearly differentiated zones above 2000 m: alpine and glacial zones [34,35]; alpine, frozen, and glacial zones [36]; or more recently, altiméditerranéen and oroméditerranéen [37] stages; montagnard méditerranéen, oroméditerranéen, and altiméditerranéen stages [38]; and oro-Mediterranean and cryoro-Mediterranean bioclimatic stages [39,40,41,42]. That is, altitudinal variations involve variations in floristic composition and distribution, which are reflected in the type and relative abundance of species present. Survival at different altitudes is therefore expected to reflect the specificity of each taxon to each altitude. 

Sierra Nevada is singular in its wide floristic diversity [43], with a special concentration of endemic species in the Mediterranean region [5], which makes it a pre-eminent biodiversity hotspot. The reasons for this high concentration of plant species are well known, as is its close association with endangered species [44]. One of the most important reasons has to do with the area’s function as a seasonal refuge in both cold (glaciation) and warm periods. The chorological spectrum of the species that inhabit these high altitudes, where alpine, arctic–alpine, Eurasian, and Holarctic species increase in importance, differs in proportion to what appears at lower altitudes [45]. This may be related to behaviors being better adapted to the snow dynamics that are very dominant in these regions, or because the exclusively endemic taxa may also show certain adaptations to such dynamics.

In view of the above, it is evident that there are uncertainties about the effects that the permanence of snow has on an early life history stage (seedlings) of restoration actions. Specifically, this could have a great impact on the survival of a wide range of species considered in conservation and recovery plans for the flora of the Mediterranean high peaks, which are particularly sensitive in this sense due to their summer drought, and the recurring and increasingly frequent periods of dryness. Therefore, the objective of this work is to analyze the global effect that the snow cover can have on the survival of seedlings of a wide range of one-year-old species (or those less than two years old) located in the Mediterranean high mountains. In addition, other variables that are scarcely explored or linked in some way to snow permanence are tested, such as altitude, edaphology, distribution range, solar radiation, and plant community. All the analyses and information were collected under the Flora Recovery Program in the High Peaks of Andalusia in SE Spain [46], and with the intention of obtaining useful results for the management of endemic species in similar environmental mountainous areas.

The work was carried out in Sierra Nevada National Park (protected area), which has the most restrictive declaration of natural spaces in Spain, and regulations on the performance of very restrictive activities, such as some agricultural and livestock activities. For this reason, these variables were been included in the work. However, we wanted to give special emphasis to the biotic factors that can have a greater impact on the survival of the seedlings. This mountain system is more than 1000 km away from any other with similar conditions (the Pyrenees, N Iberian Peninsula, and N Morocco), which translates into a high rate of exclusivity and endemicity in its flora and vegetation.

## 2. Results

**Figure 1:**
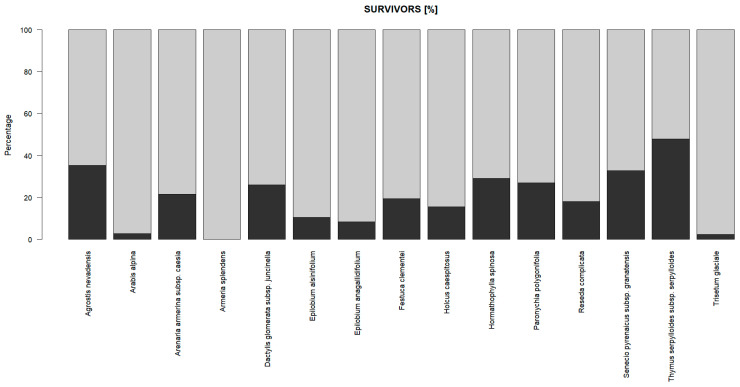
Scatterplot with survival rates (dark grey) in percentages.

Starting from a set of 15 taxa from eight families, with two life forms (chamephytes and hemicryptophytes) belonging to seven plant communities, as an overall result, a total of up to 24.53% of surviving plants (Figure 1) with 950 individuals in total surviving out of the 3873 planted was observed. The best survival rates (Table S1) were obtained by three taxa (*Plantago holosteum*, *Thymus serpylloides* subsp. *serpylloides*, and *Arenaria pungens* subsp. *pungens*), which achieved results above 60%. On the contrary, *Trisetum glaciale*, *Arabis alpina*, and *Epilobium anagallidifolium* did not reach 10%, considering an overall average result of 29.76 ± 18.19%. Regarding the rest of the characteristics of these species, there was no pattern that differentiated the groups from the rest (plot, snow cover, etc.), either in the groups of the greatest success or those of the greatest failure. The only characteristic shared by the most successful species, along with some others in the middle zone of the table, was biotype (pulvinular chamaephytes). The second year after planting, the monitored data presented very low losses compared with those for the first year monitored. However, the acclimatization process observed was so rapid that it was difficult to distinguish planted specimens from other spontaneous ones or from those that already inhabited the stand.

**Figure 2:**
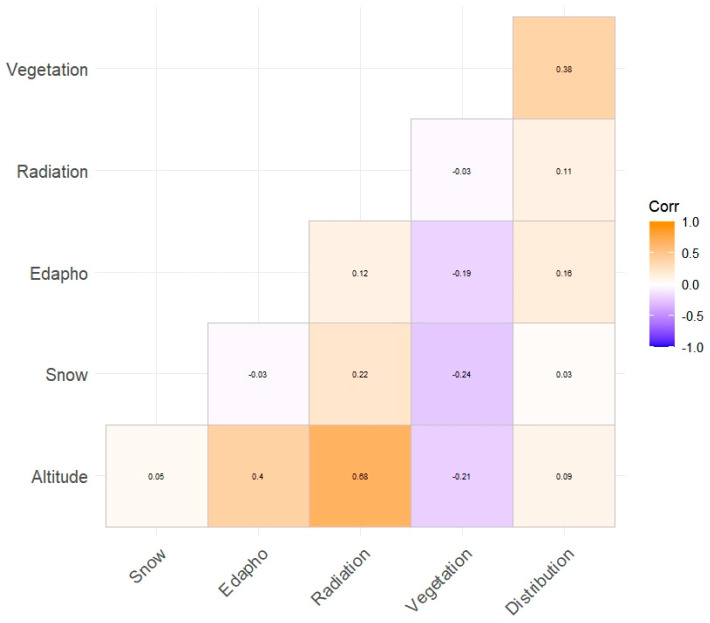
Correlations between variables involved in the GLM analysis, containing Spearman’s correlation coefficients. Altitude: altitude; Snow: snow cover permanence; Edafo: edaphology of the ground; Radiation: peak solar time in MJ·m^−2^; Vegetation.: plant community; Distribution.: plant distribution area.

In order to know the normality of the sample, the Kolmogorov–Smirnov–Lilliefors test with *n* > 50 homogeneity was applied. The results showed foreseeable non-normality in the distribution of the data of continuous variables (Table 1). For the discrete variables (χ^2^), the general results vary from moderate evidence of non-normality to great significance but with a predominance of non-normality (Table 1). In addition, with the previous results obtained, a collinearity test (Spearman’s test) was performed. (Figure 2). As expected, this only presented a moderate correlation of radiation with altitude (0.68).

**Figure 3:**
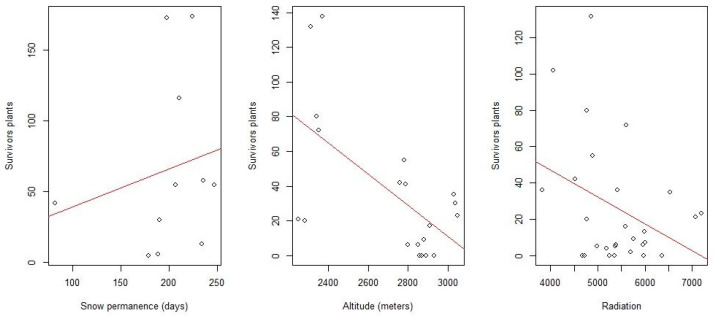
Scatterplots of survivors with trend lines in red. From left to right: snow permanence (days), altitude (meters) and radiation (MJ·m^−2^).

The χ^2^ tests were performed and revealed significance in all cases (Table 2). In some cases, there even seems to be signs of bias, as the results fall short of trends (Figure 3 and Figure A2). Furthermore, all the variables analyzed with a binomial generalized linear model (GLM) had significant results (Table 3 and Table 4).

In the global results of the survival seedlings (Table 2), it is striking that the grass species used did not stand out with the best results, with one of the species having the worst results of the entire experiment. The limited success observed in two species, *Trisetum glaciale* (exclusive to Sierra Nevada) and *Arabis alpina* (Holarctic distribution), is also interesting. For both species, it is typical to find them in the highest peaks of the Sierra Nevada. This result could be an indication of the vulnerability of these species to global warming, so it would be convenient to follow their presence and evolution in Sierra Nevada in greater detail and depth in the coming years.

**Figure A1:**
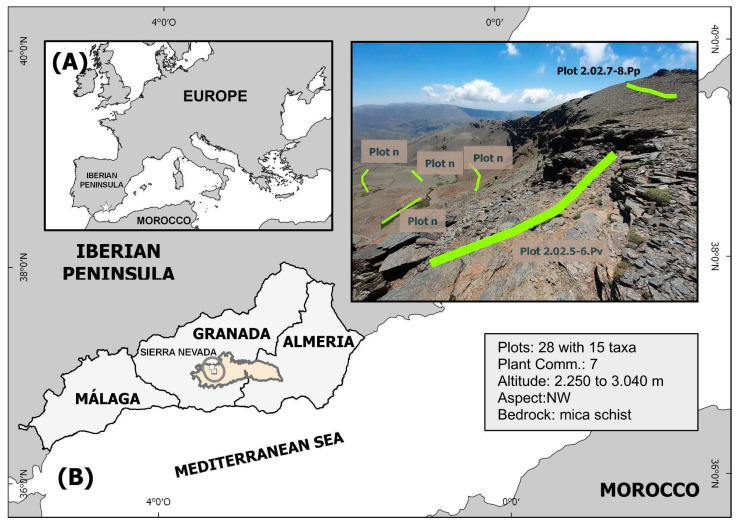
Map of study location. (**A**) Location in the south of Europe (star); (**B**) Location in the south of Spain (circle).

The results of the GLM analyses of the variables were homogeneous and very significant except in the case of plant community (Table 3 and Table A1, Figure A1), providing the chosen model with an explanation for 26.32% of the survival of the plantings carried out (residual deviance = 2620.786; null deviance = 3557.20).

Analyzing these results in detail and observing the significance of the levels of each variable, great significance can be seen in many, but not all, of these levels (Table 4). Looking at the individual data and their factorial coefficients, a satisfactory degree of significance is obtained in general (Table 3 and Table 4). The continuous variables (altitude and snow) are both highly significant (*p* < 0.001), although with values of different signs. The altitude does not seem to facilitate the survival of new seedlings; the estimated parameter is very low (−0.00276; *p* < 0.001), so this fact reduces the relative importance of this variable. On the other hand, the permanence of snow appears with a positive sign and to be of a higher order (0.02445; *p* < 0.001), thus favoring the survival chances of seedlings.

Edaphology (Table 4) presents significance in only one level (stones and rocks: −3.39597; *p* < 0.001), though globally in the model it presents significance and enriches it, improving its overall result.

For radiation, it is difficult to find ecological meaning in these results. It presents some significant factors and others without significance (discrete variable). The maximum estimated coefficient (6.11479; *p* < 0.001) coincides with the lowest radiation value (4514 MJ·m^−2^). When examining this in detail, it can be seen that the levels of this variable with significance and positive indices are grouped with the lowest values of the variable (4514–5410 MJ·m^−2^), while those with negative indices only appear in the group with the highest values (5595–5981 MJ·m^−2^). However, they appear accompanied by others that are also positive at even higher levels (5955–5981 MJ·m^−2^), which is only enough to indicate greater survival with the lowest levels of radiation within the spectrum collected.

The plant community variable did not present significance in any of the seven types/levels considered (Table 3 and Table 4). However, its integration improves the result of the model (better AIC) but without global significance (*p* > 0.1). Therefore, it is the only variable of all the variables explored that does not seem to present any type of direct link to survival.

Finally, in relation to the five distribution ranges considered in the taxa used, significance is observed in all of them (*p* < 0.01 and *p* < 0.05) except for the widest one (Amp.). Those whose distributions cover areas from the Mediterranean to Sierra Nevada show a positive index, and the highest was that of Sierras Béticas (Baetic). However, the factor referring to boreo-alpine taxa appears to be negative with a lower degree of significance.

## 3. Discussion

This work analyzed the effect of the snowpacks and other variables, which until now had not been frequently considered in studies of the reintroduction of seedlings in high mountains, on the survival of plantations in restoration trials. Our results confirm that the permanence of snow was one important factor to take into account in the studies on the survival of the plantings carried out in these Mediterranean high-mountain environments. This fact, by itself, invites us to think about the importance of this same factor in the natural dynamics of these species and its limitations in the face of the increasing absence of snow even in the winter period (meanwhile, its presence is increasingly reduced in spring). Likewise, these results are valid references with which to guide both future experiments and the next initiatives for the management of flora and/or vegetation in high peaks, particularly in the recovery plans for threatened species (threatened flora recovery plans).

The GLM analysis and the independence test showed the importance of snow permanence as a factor for survival in Mediterranean high mountain plant restoration [47]. This environmental variable has usually been obviated in plant restoration in the Mediterranean region, despite the fact that its effect on mountain plants has been well known for a long time [48]. According to this experience, plantings do better the longer the snow cover lasts on them. In other words, this high-mountain flora needs to be covered by snow during winter time. Therefore, it supports the starting hypothesis regarding the need for a longer permanence of snow to improve the results of this type of action. These results are consistent with those of other studies developed in arctic and alpine environments [49]. In restorations and other adaptive management actions, it is of great interest to have these data when planning and deciding on locations for actions of this type. With this information, the ultimate location of actions can be chosen with a clear advantage for survival and the maximization of limited resources.

**Figure A2:**
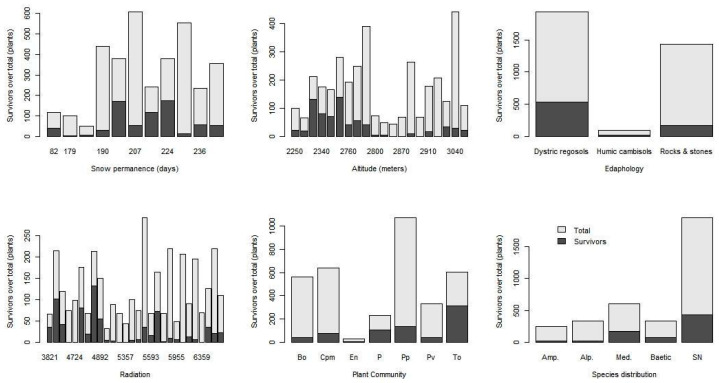
Histogram of survivors (dark gray) versus total plants planted (light gray) in relation to the variables considered. Radiation: 10 m accuracy in data in MJ·m^−2^. Plant community: mire (Bo); scree vegetation (dwarf shrub) (Cpm); shrub (juniper) (En); *Festuca* pastures (grassland) (P); psycro-xerophilous pastures (grassland) (Pp); snowdrift grasslands (Pv); dwarf shrubs (To). Species distribution: wide distribution (Amp.); alpine species (Alp.); Mediterranean region (Med.); Baetic ranges (Baetic); Sierra Nevada s.l.1 (SN).

The result obtained regarding altitude indicated an overall negative relationship between this variable and survival, since it was observed that as elevation increases, survival worsens. This may seem contradictory since the higher the altitude, the longer the snow remains. However, it must also be taken into account that as you ascend, the weather conditions also become harder with stronger winds, hard rime, extreme temperatures, and insolation, above all. Observing each taxon separately, higher survival rates are seen to coincide with the ranges where their greatest abundance currently occurs (Figure 3 and Figure A2). However, a noticeable increase is perceived at high altitudes that could be associated with places where snow cover is preserved for a longer time.

With less snow cover to protect plants, this aspect could be an indicator of an increase in the risk of extinction of those species that are forced to ascend in altitude due to climate change phenomena, since when they are forced to ascend, they find harsher environmental conditions that decrease their survival, and it is expected that this will influence their population numbers in the long term [19]. 

The edaphic variable only shows clear significance in one of its typologies (stones and rocks), which is also negative. Therefore, it could be deduced that the edaphic type does not appear to have an influence in terms of survival, except for on the habitat of stones and rocks. This habitat yields the highest values and a negative sign in the analysis, and is therefore the most complex when it comes to implementing plant colonization.

With regard to radiation, a detailed observation of the factors does not show a clear grouping of negative or positive values (lower or higher survival, respectively). Higher radiation levels (inside that variable) are the ones that appear most frequently as negative values (Table 4). However, there are some (with low levels) that are positive, so the statement that high radiation harms the planted seedlings is not very robust. The results suggest that this variable is interrelated with some other variable not studied, and thus it is difficult to discern its true effect. It may be that this result is due to the type of data (discrete variables) and low precision in the radiation starting data. This also occurs with other meteorological variables in the Sierra Nevada environment due to its complicated topography. 

When globally analyzing the native vegetation, it was observed that there is a great link with snow cover regardless of the plant community. despite the fact that, a priori, it might be expected that some communities would show greater dependence on this permanence (both positive and negative snow permanence time). However, the scant significance found in the plant community variable could be justified by two factors: (1) the effect of the grazing stock in the study area as a “distorting element” in the system, in addition to its more-than-possible preference for certain places, and (2) the little differentiation at the ecological level (with these variables) between the different communities (despite their physiognomic differences) existing at these heights in terms of colonization/survival in the early stages.

One of the most surprising results of the study is related to the distribution range variable. Taking into account the relatively limited pool of taxa used in the study, it is worth highlighting the fact that the most exclusive taxa present, by far, had the highest survival rates. This invites us to think that these plants are better adapted to the particular conditions of the Mediterranean high mountains, while those with a wider distribution do not show any advantage or disadvantage (without significance). Perhaps the most remarkable finding is the unfavorable results of the boreo-alpine species. These differences could be explained by the adaptations of endemic species that are morphologically and physiologically better prepared for the unique conditions of the Mediterranean high mountains (occasional heat waves, extreme temperatures, years without snow, etc.). With this in mind, it can be deduced that under equal conditions, more endemic Mediterranean seedlings, even those with wide distributions, survive better than do the boreo-alpine ones. Therefore, species with a boreo-alpine distribution that reach this mountain range as the extreme southern limit of their distributions would be comparatively more threatened. This result has a significant implication in the management and implementation of conservation measures. In Spain, it is common for legislation and recovery plans to give more importance to endemic plants than the rest. However, based on this result, it would be necessary to review both the protection catalogs and the priority action measures within and/or outside of the recovery and conservation plans. This is reflected in the lists of threatened local flora, which are basically species in these two groups (species at their distribution limit and endemic species) [50,51]. All of the above justifies special attention to these two groups in particular.

Although these results are in line with those of the previous research, which is cited and has results that agree with those of this study, perhaps the measurement of recorded success or failure should be considered with two limitations: (1) that effective monitoring was carried out for one year, and (2) that certain determining factors were not registered in this experiment, such as the palatability of each taxon or the grazing load in each planting sector. It is true that, being in the same basin, it could be assumed that this load would be more or less equivalent for all taxa, and not very related to the prolongation (in time) of that same load. Still, this aspect has sufficient importance for another independent and further experiment. Bearing this in mind, it would not be too risky to assume that the greatest successes were obtained by species best adapted to current climatic conditions in a scenario of global change.

To maximize restoration success in these plant communities, the observation of seedlings is recommended instead of seed germination, since the former has been confirmed to be one of the most critical stages in the life history of plant populations [52], although this stage should not be generalized to all cases, since there are specific taxa (including families such as Poaceae) that have shown excellent results after planting, as is also the case of taxa specializing in vertical/extraplum rock habitats, or, obviously, of therophytes. The rest of the variables would depend on the goals pursued, but in general, the most success can be expected at lower altitudes, with high snow permanence, and outside areas that are especially rocky or have large blocks. 

This result could be considered to be in agreement with that of other studies [47,53], except for the differences in both the number and intensity of snowfalls and in snow permanence, which are usually lower at these latitudes (Mediterranean region). In a climate scenario with less snow, the plants in it could tend to diminish as well. This hypothesis is supported by the results of other studies [7], which note that the Mediterranean region shows a progressive impoverishment of high-mountain species, and a rising trend at the optimum habitat altitude that would result in their increased scarcity [54,55,56]. 

In the design of future studies with similar topics, the particular effects of snow (its depth, permanence, etc.) on the various species should be dealt with in greater depth to confirm general and specific trends. Some species could benefit, and others may be harmed, as observed in similar environments [21]. It would also be more efficient to be able to count on other snow monitoring tools, such as fixed cameras [57] or a physically based, distributed hydrological model [58].

Managers of protected areas such as the National Park of Sierra Nevada require globally applied tools because they are usually limited in terms of time and financial resources. On the other hand, the deployment of resources necessary for an approach for each species separately, although necessary, collides head-on with truly applicable management.

## 4. Materials and Methods

Sierra Nevada is an important center of plant diversity, with 2348 described taxa [59] and a high diversity of ecological conditions in which to house the aforementioned biodiverse taxa. The work area is considered a hotspot [43], with a high richness of endemic taxa, many of them considered to be in a risk category [45]. There are also numerous species found in their southern distribution limits. Sierra Nevada is located in the SE Iberian Peninsula and, despite its proximity to the Mediterranean Sea, it has an altitudinal range from approximately 600 to 3479 m above sea level (Mulhacén I Peak) [60]. It covers an area of about 2000 km^2^, and is approximately 90 km in length from east to west. Its annual precipitation varies greatly, being very irregular with values between 350 and 1200 mm per year, depending on altitude. The average temperatures in mountain areas (2500 m) is below 0 °C during winter, at least 5 months a year. In winter, snow cover can persist for up to 8 months in the highest areas, and occasionally for up to 10 months in small and protected areas.

However, in the last decade, snow cover has decreased dramatically in both coverage and permanence, but in recent years, an increase in the average annual temperature has been observed that has been accompanied by repeated heat waves, even during normal times of low temperatures. All of this is accompanied by a noticeable drop in rainfall. As it is a Mediterranean region, the summer drought period usually lasts 4 months, although in recent years there have been periods of up to 8 months without rain (State Meteorological Agency, Madrid, Spain). 

### 4.1. Selection of Species

The choice of taxa was made based on the floristic composition existing in the different communities of the study area (Table 5 and Table A1), with the intention of testing the global restoration success of the most common communities found in summits [61]. Those taxa with good representation in the plant communities and greater accessibility for both collection and propagation were selected.

We decided to use seedlings instead of seeds, since similar experiences with seeds gave very low results in germination and survival after one year [62]. To obtain the necessary seedlings for the experiment (in 2010 and 2011), seeds were collected in the same environment defined for the plots during the summer of the previous year (2009). The collected seeds were deposited in the Plant Propagation Laboratory (Seville, Consejería de Sostenibilidad, Medio Ambiente y Economía Azul), where they were kept at 4–5 °C and under conditions of humidity below 5%, in accordance with the recommendations for seed banks [63], until their use. Those used for plant production were germinated and kept under nursery conditions for one year, then transferred to a hardening station at 1980 m (Hoya de Pedraza Botanical Garden, Sierra Nevada, Granada; Andalusian Botanical Gardens in Natural Spaces Network) where they remained for at least two months before being planted in the field. In this way, the seedlings are subjected to drought hardening, which will improve their chances of survival against freezes and droughts in the field [64]. All seedlings used were of a minimum age of one year old.

Planting was chosen since it is the usual method used in restoration in the area. The plantings were distributed along an altitudinal gradient from 2250 to 3040 masl. The plants were integrated into the original community via holes and/or areas with a scarcity of these same taxa. In total, 3873 individuals (15 taxa from 8 families) pertaining to seven plant communities were used (Table 5 and Table A1) with a mean of 19.41, and a standard deviation (SD) of 12.16 repetitions of plants per linear plot (in 28 plots). The plantings were carried out in autumn 2010, from 21 October to 2 November, and their follow up was carried out during the following summer, in 2011, from 1 of July to 17 August, and the summer of 2012, from the 1 August to 10 August.

For taxonomic considerations, the latest revision of flora of the SE Iberian Peninsula [65] was followed for data on the biology, taxonomy, and spatial distribution variables of each species. The pool of taxa included only two life forms: Chamaephyte and Hemicryptophyte. The therophytes were excluded, as they introduced a greater degree of complexity to the experiment, due to the large number of added variables that are difficult to control (in terms of the difficulty in distinguishing them, the germination percentage, asynchronous germination, etc.). In order to obtain more general and less species-specific conclusions, a more representative sample was collected by avoiding taxa that are especially threatened, since these can yield very biased results as a consequence of them presenting added difficulties (such as limited reproductive biology, extremely low population numbers, uncontrolled threats, etc.). No distinctions were made between those with clonal reproduction for two reasons: firstly because there is evidence of seedling recruitment in this type of species with clonal reproduction [66]; secondly because when environmental conditions worsen, a noticeable drop in sexual reproductive potential is observed [67,68]. Therefore, it is highly recommended to encourage the generation of new individuals through sexual reproduction in these high-mountain environments, especially aiming at an adaptive management response to global warming.

### 4.2. Selection of Experimental Sites and Variables

**Figure 4:**
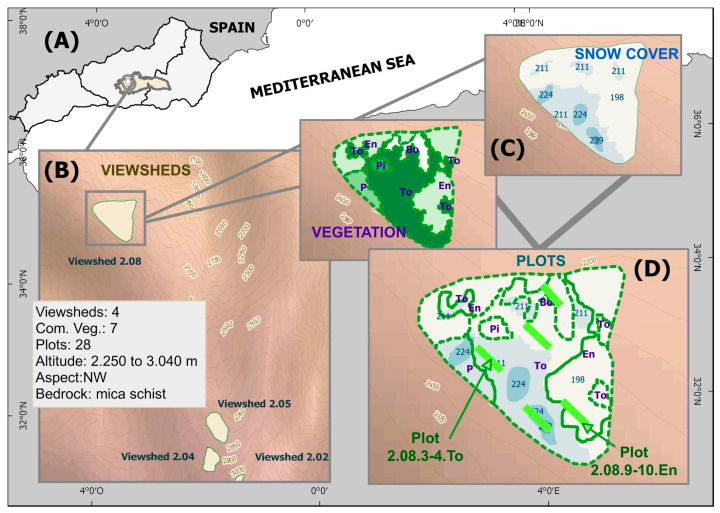
(**A**) Map of study location where plants were located in Spain, SE Iberian Peninsula (square). (**B**) Map with viewshed limits in Sierra Nevada National Park. (**C**) Schematic representation of variables used (in green and blue respectively) to generate final plots. (**D**) Parcel location scheme in green with variables.

Experiments on high-mountain (2250–3050 m) plant communities were carried out in the highest areas of Sierra Nevada (Spain) during the summers of 2010 and 2011. A field experimental design was used; planting was conducted on 28 linear plots with variable lengths depending on the number of available plants (seedling, length: 14.71 SD 6.13 m, width: 2.50 SD 1.59 m; sowing, length: 1.25 SD 0.57 m, width: 1.92 SD 1.68 m) distributed throughout the study area (Figure 4, Table S1), which were located at different altitudes and encompassed a gradient of the different environmental variables considered. Each taxon is located in the same type of plant community where it was collected, consistent with the bibliography [69]. The nomenclature followed to identify each plot is a combination of the following elements: (1) A viewshed (one among four, named from 2.02 to 2.08)—the viewsheds cover large surfaces with great differences between the different variables analyzed (a difference from 60 m to 220 m in altitude depending on the basin); (2) a linear planting plot with the start and end points marked, always following the same altitudinal level (1–2, 3–4, 5–6, and so on depending on plant availability); and (3) the plant community where it is located (e.g., “Bo” for Mire vegetation, Table 5). These were identified in such a way that all the plots were named as following the example (2.02.1-2.Bo) and always had variations in at least one of the variables considered in the study.

The plots were established in zones with homogeneous ecological characteristics in order to minimize the number of factors relating to other variables outside the objectives of this study, thus avoiding deviations in the results. The coverage of herbaceous plants or other species was also taken into account so as not to harm the growth and survival of the seedlings [70,71]. There were six objective variables (Table 5): snow permanence and altitude, which are numerical and continuous variables; edaphology, distribution range, and plant community, which are categorical variables; and solar radiation, which is a numerical and discrete variable.

**Figure 5:**
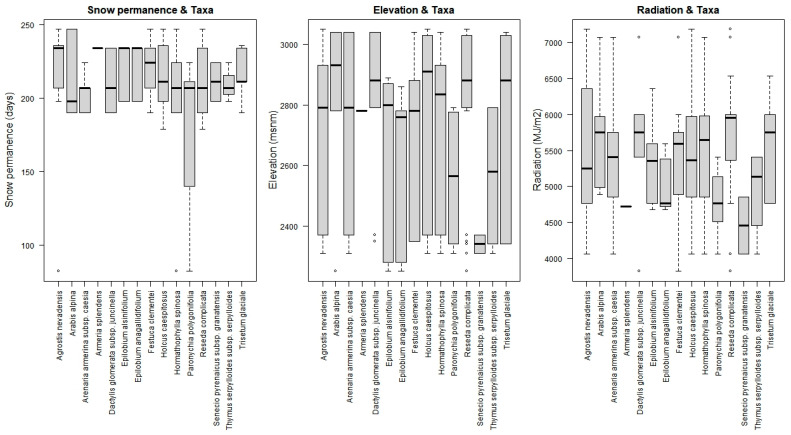
Boxplot with the plantations of each taxon, distributed across the continuous variables considered in the analysis (snow permanence, elevation, and radiation).

Snow permanence, a continuous variable, is the first variable in the study (Figure 5). This variable was analyzed via the photographic surveillance of 4 visible watersheds (four viewsheds, Figure 4), which included all linear working plots (mean area: 8.67; SD: 8.26 ha) for a full year prior to starting plantings (2010–2011 winter). Surveillance visits were performed in intervals of 15.93 SD 3.53 days according to the meteorological events that occurred (e.g., rainfall in the form of storms, snowfall, strong winds, etc.). The result of that single year of monitoring is assumed as a standard measure based on other examples of the periodic monitoring of snow cover during previous and subsequent years [72,73]. It was observed that the dominant winds were from the SW in the Sierra Nevada Mountains. Thus, snow packs, conditioned by this factor along with topography, accumulated in the same zones throughout the study, as well as in others years. This persistence of snowpack in spring and summer was also observed in the same places, in agreement with the results of other studies [74] where no significant interannual variations were perceived for four years (1998–2002) in those locations where snowpack accumulates. 

Taking topographic environmental data from the basic 1:10,000 scale map [75], altitude was another continuous variable, not counting lower altitudes where the presence of snow is insignificant during winter and spring (2250 to 3050 m).

For soil characterization (edaphology), data from the basic 1:100,000 scale map [76] were used. This categorical variable was later confirmed through in situ field visits to each plot. Three levels were found: humic cambisols, dystric regosols, and rocks and stones.

The distribution range of a taxon is the area of a taxon’s occurrence. In the case of Sierra Nevada, practically every taxon has a different distribution range [65]. There are up to 13 different distributions, which on many occasions are due to small differences (Table A1). To be able to make comparisons, they were simplified into the following five categories: widely distributed species; species distributed mainly in the Mediterranean region; species that are alpine, boreo-alpine, circumboreal, or Holartic in the wide sense; species with distributions mainly limited to the Baetic ranges; and species with distributions exclusively or nearly exclusively within the study area (Sierra Nevada).

The mean annual total radiation (in MJ·m^−2^) in a cartographic format and as a discrete variable [77] was based on the calculation of direct radiation and diffuse circumsolar radiation for every 10 × 10 square meters. A mathematical model of the atmosphere was used in which the different parameters used (temperature, turbidity, optical indices, etc.) were calibrated using data measured at stations. The data in the planting zone span 28 different levels (3821 to 7186 MJ·m^−2^). The data format was obtained as a discrete variable. 

The data on plant community types (categorical variable) were taken directly from each plot. Seven plant communities were differentiated (Table 5) via the detailed mapping of vegetation. This initially followed the methodology applied in the Sierra Nevada Mountains by Molero et al. [69], based on cartographic surveys through photo-interpretation and an inventory of the floristic taxa and vegetation using phytosociological methods. The most detailed methodology regarding the plant communities described in the work area has been reviewed by several authors [69,78,79,80]. However, the extremely high differentiation between plant communities makes their comparison difficult, and thus we opted for simplification according to the physiognomic types: mire (Bo); scree vegetation (dwarf shrub) (Cpm); shrub (juniper) (En); *Festuca* pastures (grassland) (P); psycro-xerophilous pastures (grassland) (Pp); snowdrift grasslands (Pv); dwarf shrubs (To).

QGis software 3.12 [81] was used to implement all cartographic information (along with planting locations, radiation, altitude, and edaphology).

### 4.3. Statistical Analysis

In order to calculate the survival rates of the plantings, the data related to the planting carried out in 2010 (for 12 days in autumn, before the first snowfall) were taken in 2011 (in the middle of summer, 8 months later). The data only consider survival (a binary variable) without going into detail regarding the degree of development. 

Before any other statistical testing, a homogeneity test was conducted in order to confirm the foreseeable non-normality in the distribution of the data of continuous variables (Kolmogorov–Smirnov–Lilliefors test with *n* > 50). The six chosen variables (snow, altitude, edaphology, plant community, radiation and distribution range) were tested separately to see their significance with respect to survival (living seedling or dead seedling) using a χ^2^ test. Only for this test were the continuous variables (snow and altitude) transformed into discrete variables, and they were considered to be categorical, assuming each value as a level of said variable. For snow cover, transformation was achieved by establishing as many 15-day levels as possible in accordance with the original sampling design (11 levels in total). For altitude, sections of 100 m (8 levels in total) were considered. The purpose was to be able to apply the same type of analysis to all the variables and allow a comparison between all of them. 

Before continuing, the existing collinearity between all the selected variables was tested using Spearman’s correlation test. This allowed us to better evaluate the results of the following analysis.

Then, with all the variables together, an analysis conducted by fitting generalized linear models (GLMs) [82], which allowed the significance of each variable to be revealed, was performed. The use of GLMs has been previously validated as an ecological data analysis tool [83], where the normality and homoscedasticity of the data are often not met. Also in the case of variables categorical, all levels to be tested were used for determining the relevance of each variable at all levels. The model used all the cited variables. The data were grouped based on variables corresponding to surviving and deceased individuals within each plot. Since the considered response variable was binomial (dead or alive plant; 0 or 1), survival followed a binomial distribution model, a binary response in the data with a logit link function. R software version 4.3.1 [84] was used for all the statistical analyses with a level of significance of *p* ≤ 0.001 in the independence and homogeneity tests (the Kolmogorov–Smirnof–Liliefors test). This method was chosen because the data are not continuous in all its values. Not all levels have the same plant communities the same species in all the plots, the same time of snow permanence in all the cases, etc. With this method, it was possible to complement the previous one (χ^2^) and the effect of all the variables together having a global response.

## 5. Conclusions

In view of the results obtained in this work, it can be concluded that the permanence of snow in the Mediterranean high mountains is a crucial factor to take into account, especially with regard to the design of management measures in these habitats (restoration, recovery and conservation plans, the management of protected natural areas, etc.). When considering restoration actions, the location of snowpacks must be taken into account, and the most rocky and stony environments should be avoided, as they make it difficult for the seedlings to survive. Promoting the use of boreo-alpine species may ensure their continuity over time despite the obtention of less successful results overall.

Those species that are forced to migrate to areas of higher elevation are expected to lose more specimens in the process due to the hardening of the conditions for establishment (like *Trisetum glaciale* or *Arabis alpina*), in addition to the decrease in potential habitat as there is less surface area in general. In the design of plans and catalogs for the protection of threatened flora, it is recommended that greater weight be given to those taxa that, without being exclusive to the local environment, reach the southernmost extreme limit of their global distribution. This is because, to date, they have been ignored in comparison with the endemic taxa of the region as they lack this taxonomic exclusivity.

## Figures and Tables

**Table 1:** Results of normality tests with the Kolmogorov–Smirnov–Lilliefors test for continuous variables (snow and altitude) and of the χ^2^ tests for the rest of the variables.

Variable	Normality	
Snow	0.17564	***
Altitude	0.25439	***
Edaphology	426.47	·
Plant Community	3690.3	*
Radiation	15,701	***
Distribution Range	1531.9	·

Significance level: ***, *p* < 0.001; *, *p* < 0.005; ·, *p* > 0.1.

**Table 2:** Results of χ^2^ tests between variables and survival results.

Variable	χ^2^ Tests	
Snow	649.5425, df = 10	***
Altitude	728.9778, df = 19	***
Edaphology	114.3517, df = 2	***
Plant Community	85.31395, df = 4	***
Radiation	820.7352, df = 27	***
Distribution Range	591.4444; df = 6	***

Significance level: ***, *p* < 0.001.

**Table 3:** The significance of each variable in the results of the analysis of variance in the GLM with seedling survival (***, *p* < 0.001; **, *p* < 0.01; -, no significance); binomial error distribution family and the canonical link function (logit).

Variable	Df	Deviance	Resid. Df	Resid. Dev	
NULL			3458	3557.168008	-
Altitude	1	479.069	3457	3078.098671	***
Snow	1	10.032	3456	3068.066032	**
Edaphology	2	51.033	3454	3017.032695	***
Radiation	23	349.725	3431	2667.307451	***
Plant Community	7	217.1368	4416	4212.44011	-
Distribution	4	46.521	3427	2620.786436	***

**Table 4:** Results of the GLM binomial link, only showing the significance level (up to *p* < 0.1). Altitude: continuous variable. Snow: continuous variable. Edaphology: discrete variable. Radiation: discrete variable with the value in MJ·m^−2^. Distribution: discrete variable; wide distribution (Amp.) that is included in the intercept level. Alpine species (Alp.); Mediterranean region (Med.); Baetic ranges (Baetic); Sierra Nevada s.l. (SN).

	Estimate	Std. Error	z Value	
(Intercept)	0.55799	1.77291	0.31473	
Altitude	−0.00276	0.00045	−6.15907	***
Snow	0.02445	0.00739	3.30855	***
Edaphology: Stones and Rocks	−3.39597	0.86870	−3.90925	***
Radiation: 4514	6.11479	1.21077	5.05031	***
Radiation: 4855	0.57840	0.32850	1.76073	·
Radiation: 4892	2.74985	0.44423	6.19013	***
Radiation: 5177	1.27587	0.68270	1.86885	·
Radiation: 5386	2.73445	0.84363	3.24129	***
Radiation: 5410	1.68823	0.46968	3.59439	***
Radiation: 5595	−0.71829	0.32168	−2.23293	*
Radiation: 5755	−0.87135	0.41315	−2.10906	*
Radiation: 5955	2.63749	0.68570	3.84639	***
Radiation: 5981	2.40640	0.53763	4.47590	***
Distribution: Alp.	−0.75441	0.36463	−2.06897	*
Distribution: Med.	1.80713	0.68356	2.64369	**
Distribution: Baetic	2.14136	0.69423	3.08449	**
Distribution: SN	1.46485	0.67081	2.18372	*

Significance level: ***, *p* < 0.001; **, *p* < 0.01; *, *p* < 0.05; ·, *p* < 0.1.

**Table 5:** Summary of the variables considered.

	Data Levels and Range	Units
Snow	82–247	days
Altitude	2250–3050	meters
Edaphology	Dystric regosols, humic cambisols, stones and rocks	type
Plant Community	Mire (Bo); scree vegetation (dwarf shrub) ^2^ (Cpm); shrub (juniper) (En); *Festuca* pastures (grassland) (P); psycro-xerophilous pastures (grassland) (Pp); snowdrift grasslands (Pv); dwarf shrubs (To)	type
Radiation	3821–7365 (10 m accuracy in data)	MJ·m^−2^
Distribution Range	Wide distribution (Amp.); alpine species (Alp); Mediterranean region (Med.); Baetic ranges (Baetic); Sierra Nevada s.l. ^1^ (SN)	areas

^1^ Sierra Nevada s.l. refers to the taxa that inhabit both the Nevada massif exclusively and those whose distribution occasionally escapes to some neighboring mountain range. More information about distribution range can be found in Table A1. ^2^ Scree vegetation remains physiognomically like dwarf shrub but its floristic composition is clearly different.

**Figure A3:**
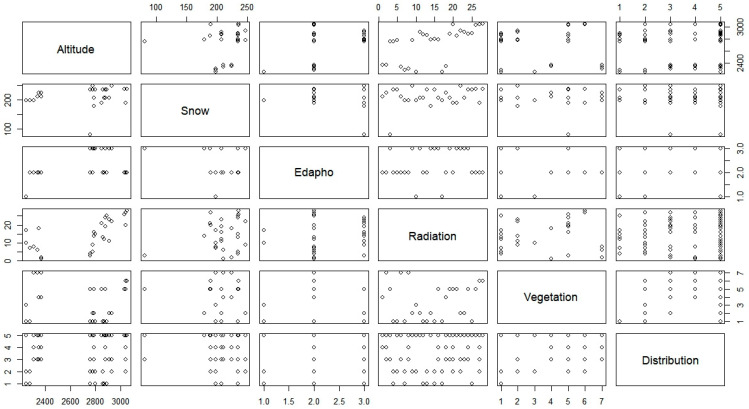
Multi-panel scatterplot of variables involved in the GLM analysis. The upper-right panels show pairwise scatterplots between each variable. Altitude: altitude in meters; Snow: snow permanence; Edafo: edaphology of the ground; Radiation: peak solar time in MJ·m^−2^; Vegetation: plant community; Distribution: plant distribution area.

**Figure A4:**
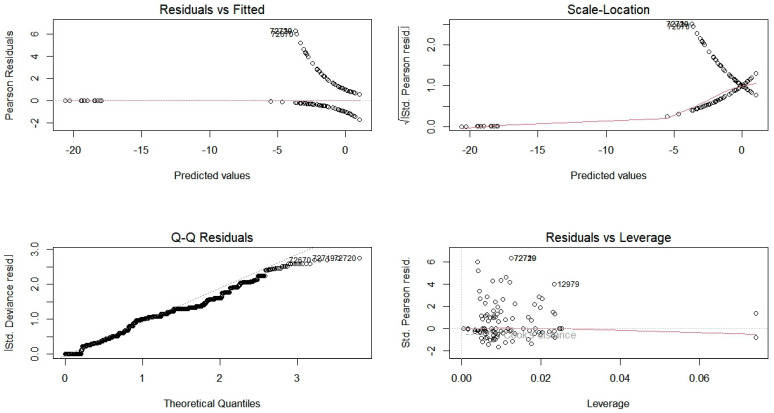
Graphical result of the residuals of the GLM analysis. Standardized residuals against predicted values (top left); probability plot of normality (bottom left) and outliers (bottom right).

## Data Availability

The data presented in this study are available on request from the corresponding author. The data are not publicly available due to the original data comes from the administration responsible for the environment (Government) and is provided upon request. The access structure is complex and authors can facilitate its access and understanding.

## Supplementary Materials

The following supporting information can be downloaded at https://www.mdpi.com/article/10.3390/plants13060783/s1: Table S1: List of the 15 taxa used in the analyses with additional information; Table S2: Additional information about plots and viewshed.

## Appendix B

**Table A1:** Additional information on taxa about variables in the study. Family, taxonomic family; Vegetation, plant communities where the plant lives: Mire (Bo); scree vegetation (dwarf shrub) (Cpm); shrub (juniper) (En); *Festuca* pastures (grassland) (P); psycro-xerophilous pastures (grassland) (Pp); snowdrift grasslands (Pv); dwarf shrubs (To); Br-Bl, Braun-Blanquet density/cover scores (source: bibliography); total cover, global cover of the plant community; Altitude, altitudinal level where the plots are located; Snow, number of days per year registered with snow cover; Radiation, mean annual total radiation (in MJ·m^−2^); Distribution, simplified distribution used in the model followed by the real distribution of species; Soil: edaphology type.

Taxon	Family	Vegetation	Br-Bl	Total Cover	Altitude	Snow	Radiation	Distribution	Soil
*Thymus serpylloides* Bory subsp. *serpylloides*	Labiatae	La, Pp, To	2, 1, +	50–70%	2270–2790	99–224	4058–7365	Sierra Nevada s.l. (SN): Sierra Nevada y Sierra de Filabres	Dystric regosols, Stones and rocks
*Arenaria armerina* subsp. *caesia* (Boiss.) C. Díaz, C. Morales & F. Valle	Caryophyllaceae	La, Pp, Pv, To	2, 1, +	10–70%	2270–3040	99–224	4058–7365	Baetic ranges (Baetic): Eastern Andalusia	Humic cambisols, Dystric regosols, Stones and rocks
*Senecio pyrenaicus* subsp. *granatensis* (Boiss.) Rivas Mart.	Asteraceae	La, To	2, 1, +	20–40%	2270–2370	99–224	4058–7365	Sierra Nevada s.l. (SN): Sierra Nevada, Sierra de la Sagra y Sierra de Tejeda	Humic cambisols, Dystric regosols, Stones and rocks
*Agrostis nevadensis* Boiss.	Poaceae	Pv, Pp, Bo, To, Cpm, P	+	50–65%	2310–3050	82–247	4058–7186	Sierra Nevada s.l. (SN): Sierra Nevada	Humic cambisols, Dystric regosols
*Armeria splendens* (Lag. & Rodr.) Webb	Plumbaginaceae	Bo, La	2, 1	50–90%	2270–2780	99–234	5251–7123	Sierra Nevada s.l. (SN): Sierra Nevada	Dystric regosols, Stones and rocks
*Hormathophylla spinosa* (L.) P. Küpfer	Brassicaceae	To, P, Pp, Pv, Cpm	2, 1, +	15–20%	2310–3040	82–247	4058–7074	Mediterranean region (Med.): Western Oro-Mediterranean	Humic cambisols, Dystric regosols, Stones and rocks
*Paronychia polygonifolia* (Vill.) DC.	Caryophyllaceae	Pp, To	+	30–80%	2310–2790	82–224	4058–5410	Mediterranean region (Med.)	Humic cambisols, Dystric regosols, Stones and rocks
*Dactylis glomerata* subsp. *juncinella* (Bory) Stebbins & Zohary	Poaceae	P, Pp, Pv	1, +	15–50%	2350–3040	190–234	3821–7074	Baetic ranges (Baetic): Betic-Maghrebi	Dystric regosols, Stones and rocks
*Festuca clementei* Lam.	Poaceae	P, Pp, Pv, Cpm	3, 2, 1	10–75%	2350–3040	190–247	3821–7074	Sierra Nevada s.l. (SN): Sierra Nevada	Dystric regosols, Stones and rocks
*Reseda complicata* Bory	Resedaceae	To, P, Pp, Pv, Cpm, En	3, 2, 1	10–75%	2250–3050	179–247	3821–7186	Sierra Nevada s.l. (SN): Sierra Nevada	Dystric regosols, Stones and rocks
*Holcus caespitosus* Boiss.	Poaceae	To, Pp, Pv, Cpm	1, +	2–15%	2310–2930	179–236	4058–7186	Sierra Nevada s.l. (SN): Sierra Nevada	Dystric regosols, Stones and rocks
*Epilobium alsinifolium* Vill.	Onagraceae	Bo	1, +	40–75%	2250–2890	198–234	4679–6359	Wide distribution (Amp.): European	Humic cambisols, Dystric regosols, Stones and rocks
*Epilobium anagallidifolium* Lam.	Onagraceae	Bo	+	40–75%	2250–2860	198–234	4679–5593	Alpine species (Alp.): Circumboreal	Humic cambisols, Dystric regosols
*Arabis alpina* L.	Brassicaceae	En, Cpm, Pp, Pv	1	5%	2250–3040	190–247	4892–7074	Alpine species (Alp.): Holartic	Humic cambisols, Dystric regosols, Stones and rocks
*Trisetum glaciale* (Bory) Boiss.	Poaceae	Pp, To	1, +	25–30%	2340–3040	190–236	4765–6531	Sierra Nevada s.l. (SN): Sierra Nevada	Dystric regosols, Stones and rocks
