# Supplementary material for: The Role of Snow-Related Environmental Variables in Plant Conservation Plans in the Mediterranean Mountains
Source: Plants (Basel). 2024 Mar 10;13(6):783. doi: 10.3390/plants13060783 (PMC10975130; doi:10.3390/plants13060783)
Supplement: Supplementary file 1 [file plants-13-00783-s001.zip › plants-2897683-supplementary.pdf]

Supplementary Material

The role of snow-related environmental variables in plant conservation plans in the Mediterranean Mountains

Jose A. Algarra, Paloma Cariñanos and María M. Ramos-Lorente

Table S1. List of the 15 taxa used in the analyses with additional information. Year 0 2010: number of seedlings introduced; Year 0 2011: number of surviving (with percentage); NPlots: number of total plots with each species; Plots number: original plots name.

Taxon	Year 0 2010	Year 1 2011(%)	NPlots	Plots number
Agrostis nevadensis Boiss.	402	142(35.32%)	13	2.02.5-6.Pv; 2.02.7-8.Pp; 2.04.5-6.Bo; 2.04.1-2.Bo; 2.04.13-14.Cpm; 2.05.1-2.Bo; 2.05.11-12.Cpm; 2.05.7-8.Pp; 2.05.9-10.Pp; 2.08.3-4.To; 2.08.5-6.To; 2.08.11-12.P; 2.08.7-8.To
Arabis alpina L.	142	4(2.82%)	5	2.02.3-4.Pp; 2.02.1-2.Pv; 2.05.11-12.Cpm; 2.04.13-14.Cpm; 2.08.9-10.En
Arenaria armerina subsp. *caesia* (Boiss.) C. Díaz, C. Morales & F. Valle	220	97(44.09%)	11	2.02.3-4.Pp; 2.02.1-2.Pv; 2.05.7-8.Pp; 2.08.3-4.To; 2.08.7-8.To
Armeria splendens (Lag. & Rodr.) Webb	253	80(31.62%)	7	2.05.1-2.Bo
Dactylis glomerata subsp. *juncinella* (Bory) Stebbins & Zohary	216	56(25.93%)	6	2.02.3-4.Pp; 2.02.1-2.Pv; 2.04.7-8.Pp; 2.05.7-8.Pp; 2.08.11-12.P; 2.08.13-14.P
Epilobium alsinifolium Vill.	247	26(10.53%)	7	2.04.1-2.Bo; 2.04.3-4.Bo; 2.04.5-6.Bo; 2.05.3-4.Bo; 2.05.5-6.Bo; 2.08.1-2.Bo; 2.08.15-16.Bo
Epilobium anagallidifolium Lam.	191	16(8.38%)	6	2.04.3-4.Bo; 2.05.1-2.Bo; 2.05.3-4.Bo; 2.05.5-6.Bo; 2.08.1-2.Bo; 2.08.15-16.Bo
Festuca clementei Lam.	226	44(19.47%)	7	2.02.1-2.Pv; 2.02.3-4.Pp; 2.04.7-8.Pp; 2.05.7-8.Pp; 2.05.11-12.Cpm; 2.08.11-12.P; 2.08.13-14.P
Holcus caespitosus Boiss.	469	73(15.57%)	12	2.02.1-2.Pv; 2.02.3-4.Pp; 2.02.5-6.Pv; 2.02.7-8.Pp; 2.04.11-12.Cpm; 2.04.13-14.Cpm; 2.04.15-16.Cpm; 2.05.11-12.Cpm; 2.05.13-14.Cpm; 2.08.3-4.To; 2.08.5-6.To; 2.08.7-8.To

Taxon	Year 0 2010	Year 1 2011(%)	NPlots	Plots number
Hormathophylla spinosa (L.) P. Küpfer	444	129(29.05%)	12	2.02.1-2.Pv; 2.02.3-4.Pp; 2.04.7-8.Pp; 2.04.9-10.Pp; 2.04.11-12.Cpm; 2.04.13-14.Cpm; 2.05.7-8.Pp; 2.05.9-10.Pp; 2.08.3-4.To; 2.08.5-6.To; 2.08.7-8.To; 2.08.11-12.P
Paronychia polygonifolia (Vill.) DC.	156	42(26.92%)	5	2.05.7-8.Pp; 2.05.9-10.Pp; 2.08.3-4.To; 2.08.5-6.To; 2.08.7-8.To
Reseda complicata Bory	596	107(17.95%)	18	2.02.1-2.Pv; 2.02.3-4.Pp; 2.02.5-6.Pv; 2.02.7-8.Pp; 2.04.7-8.Pp; 2.04.9-10.Pp; 2.04.11-12.Cpm; 2.04.13-14.Cpm; 2.04.15-16.Cpm; 2.04.17-18.Pp; 2.05.11-12.Cpm; 2.05.13-14.Cpm; 2.08.3-4.To; 2.08.5-6.To; 2.08.7-8.To; 2.08.9-10.En; 2.08.11-12.P; 2.08.13-14.P
Senecio pyrenaicus subsp. *granatensis* (Boiss.) Rivas Mart.	69	29(42.03%)	4	2.08.3-4.To; 2.08.7-8.To
Thymus serpylloides Bory subsp. *serpylloides*	160	103(64.38%)	5	2.05.7-8.Pp; 2.08.3-4.To; 2.08.7-8.To
Trisetum glaciale (Bory) Boiss.	82	2(2.44%)	4	2.02.3-4.Pp; 2.02.7-8.Pp; 2.04.7-8.Pp; 2.08.5-6.To

Table S2. Additional information about plots and viewshed.

Plots	Plant Community	Viewshed
2.04.1-2.Bo	Mire	2.04
2.05.1-2.Bo	Mire	2.05
2.08.1-2.Bo	Mire	2.08
2.08.15-16.Bo	Mire	2.08
2.04.3-4.Bo	Mire	2.04
2.05.3-4.Bo	Mire	2.05
2.04.5-6.Bo	Mire	2.04
2.05.5-6.Bo	Mire	2.05
2.04.11-12.Cpm	scree vegetation (Dwarf Shrub)	2.04
2.05.11-12.Cpm	scree vegetation (Dwarf Shrub)	2.05
2.04.13-14.Cpm	scree vegetation (Dwarf Shrub)	2.04
2.05.13-14.Cpm	scree vegetation (Dwarf Shrub)	2.05
2.04.15-16.Cpm	scree vegetation (Dwarf Shrub)	2.04
2.08.9-10.En	Shrub (Juniper)	2.08
2.08.11-12.P	*Festuca* pastures (Grassland)	2.08
2.08.13-14.P	*Festuca* pastures (Grassland)	2.08
2.04.17-18.Pp	Psycro-xerophilous pastures (Grassland)	2.04
2.02.3-4.Pp	Psycro-xerophilous pastures (Grassland)	2.02
2.02.7-8.Pp	Psycro-xerophilous pastures (Grassland)	2.02
2.04.7-8.Pp	Psycro-xerophilous pastures (Grassland)	2.04
2.05.7-8.Pp	Psycro-xerophilous pastures (Grassland)	2.05
2.04.9-10.Pp	Psycro-xerophilous pastures (Grassland)	2.04
2.05.9-10.Pp	Psycro-xerophilous pastures (Grassland)	2.05
2.02.1-2.Pv	Snowdrifts grasslands	2.02
2.02.5-6.Pv	Snowdrifts grasslands	2.02
2.08.3-4.To	Dwarf shrubs	2.08
2.08.5-6.To	Dwarf shrubs	2.08
2.08.7-8.To	Dwarf shrubs	2.08
